# Mapping QTL affecting resistance to Marek's disease in an F6 advanced intercross population of commercial layer chickens

**DOI:** 10.1186/1471-2164-10-20

**Published:** 2009-01-14

**Authors:** Eliyahu M Heifetz, Janet E Fulton, Neil P O'Sullivan, James A Arthur, Hans Cheng, Jing Wang, Morris Soller, Jack CM Dekkers

**Affiliations:** 1 Department of Animal Science and Center for Integrated Animal Genomics, Iowa State University, Ames, IA 50011, USA; 2 Department of Molecular Biology, Ariel University, Ariel 44837, Israel; 3 Hy-Line International, Dallas Center, IA 50063, USA; 4 USDA-ARS-ADOL, Avian Disease and Oncology Laboratory, East Lansing, MI 48823, USA; 5 Pioneer Hi-Bred International Inc., Johnston, IA 50131, USA; 6 Department of Genetics, The Hebrew University of Jerusalem, 91904 Jerusalem, Israel

## Abstract

**Background:**

Marek's disease (MD) is a T-cell lymphoma of chickens caused by the Marek's disease virus (MDV), an oncogenic avian herpesvirus. MD is a major cause of economic loss to the poultry industry and the most serious and persistent infectious disease concern. A full-sib intercross population, consisting of five independent families was generated by crossing and repeated intercrossing of two partially inbred commercial White Leghorn layer lines known to differ in genetic resistance to MD. At the F6 generation, a total of 1615 chicks were produced (98 to 248 per family) and phenotyped for MD resistance measured as survival time in days after challenge with a very virulent plus (vv+) strain of MDV.

**Results:**

QTL affecting MD resistance were identified by selective DNA pooling using a panel of 15 SNPs and 217 microsatellite markers. Since MHC blood type (BT) is known to affect MD resistance, a total of 18 independent pool pairs were constructed according to family × BT combination, with some combinations represented twice for technical reasons. Twenty-one QTL regions (QTLR) affecting post-challenge survival time were identified, distributed among 11 chromosomes (GGA1, 2, 3, 4, 5, 8, 9, 15, 18, 26 and Z), with about two-thirds of the MD resistance alleles derived from the more MD resistant parental line. Eight of the QTLR associated with MD resistance, were previously identified in a backcross (BC) mapping study with the same parental lines. Of these, 7 originated from the more resistant line, and one from the less resistant line.

**Conclusion:**

There was considerable evidence suggesting that MD resistance alleles tend to be recessive. The width of the QTLR for these QTL appeared to be reduced about two-fold in the F6 as compared to that found in the previous BC study. These results provide a firm basis for high-resolution linkage disequilibrium mapping and positional cloning of the resistance genes.

## Background

Marek's disease (MD) is a highly contagious disease that affects chickens worldwide and is caused by the Marek's disease virus (MDV), an oncogenic avian herpesvirus. MDV replicates in the lymphoid tissues [1] and is commonly manifested as an acute disease with lymphomas in multiple visceral organs [2]. MD has a large impact on production, making it the most costly viral disease in the chicken industry [3]. It has also been proposed as a natural model for lymphomas that over-express Hodgkin's disease antigen (CD30) [4], and for understanding how vaccines control lymphomas [5] and how pathogenic viruses continue to evolve [6].

The current family selection methods used for improving resistance are based on challenge exposure, and hence are very expensive in terms of selection space, and the financial and ethical cost of routinely challenging large numbers of birds. It is anticipated that identification of QTL for MD resistance will eventually allow marker-assisted selection on an individual bird level, without need for routine challenge. This will greatly enhance the accuracy of selection, and reduce costs by orders of magnitude. QTL mapping, in conjunction with positional comparative cloning, gene expression [7,8], and protein-protein interaction studies [9,10] could also provide a platform for identification of the genes underlying the QTL. The identification of these genes will provide insight on disease pathways and resistance mechanisms, leading to more effective vaccines or other control strategies, and even more effective selection schemes based on gene-assisted selection [11].

Simulation and theoretical studies [12-14] show that large scale BC or F2 experiments can locate QTL of moderate allele substitution effect (0.2 or 0.3 standardized units) only to within a confidence interval of 10 to 20 cM or more. Consequently, Darvasi and Soller [15] proposed the use of Advanced Intercross Lines (AIL) for fine mapping of QTL. An AIL is initiated by a cross between two inbred lines, and continued by random intercrossing through successive generations. Recombination events between loci accumulate with the advance of the intercross generations. The AIL approach has been applied successfully in many mouse studies (e.g., [16]). A full-sib intercross line (FSIL) [17] is a variant of the AIL that is suitable for analysis of outcrossing populations. In a FSIL, a single male and female from the same or different populations, are crossed to produce a large full-sibship. The full-sibs are mated at random, and the population continued by random intercrossing to advanced generations. To a large extent, map expansion and QTL resolution in an FSIL parallel that in an AIL.

Previous studies in commercial and non-commercial White Leghorn populations have identified genes, QTL and genomic regions associated with resistance to MD (briefly summarized in [18]; see also [19]). Heifetz et al. [18], using selective DNA pooling, identified 15 QTL affecting MD resistance in a large reciprocal backcross (BC) between two partially inbred commercial White Leghorn layer chicken lines that differed in resistance to MD. Five of these QTL were previously reported by McElroy et al. [20] in a smaller study of an independent hatch of one of these BC populations, and four were previously reported by Yonash et al. [21] in a study of an F2 population generated by a cross between two inbred White Leghorn layer lines that differed markedly in resistance to MD.

In the present study, a large F6 FSIL was generated as a continuation of the same F1 population from which the BC populations of McElroy et al. [20] and Heifetz et al. [18] were derived. The objective of the study was to confirm previously mapped QTL, improve QTL map resolution, and uncover additional QTL segregating in this population.

## Results

### Survival by family, B type and line

Table 1 shows characteristics of resistant and susceptible pools according to family and MHC blood type (BT). There was a clear effect of BT on resistance. The average proportion of birds that survived the test for Families 1, 2, 3, 5 where all blood types were represented, was 0.36, 0.41 and 0.53 for blood types B2/B2, B2/B15 and B15/B15, respectively. Similarly, the average survival time of the birds in the susceptible pools for families where all three blood types were represented was 56.8, 55.1 and 66.3 days for the B2/B2, B2/B15 and B15/B15 blood types, respectively. Thus, blood type B15/B15 was clearly more resistant, but the resistance appeared to be fully recessive, as survival of the B2/B15 heterozygote was virtually identical to that of the B2/B2 homozygote. Family effects were also apparent, with Families 1 and 2 showing higher proportions of survivors, and higher average survival times in the susceptible pools, as compared to the other three families. In the ANOVA analysis, Family (p < 0.0001) and BT (p = 0.047) were both significant. The results of the pure line chicks that were added to the experiment as contemporary controls were 28.6% mortality for Line 1 and 7.6% mortality for Line 2. This difference of 21.0% in favour of Line 2 was less than observed in previous trials, where the difference was 41.4% and 42.7% in favour of Line 2 [18]. At 56.7%, the average mortality rate was much higher in the F6 families than in the pure line chicks but close to the target mortality for the challenge test (50%).

**Table 1:** Pool composition according to family (F) and MHC type (BT)

F	BT	Total No.	Resistant		Susceptible		
			
			No.	Prop	No.	Mean	Range
1	2/15	144	30	0.51	29	59.1	30–73
	2/2	51	27	0.44	12	59.8	30–73
	2/2^1^	-	-	-	12	94.0	73–113
	15/15	96	30	0.71	19	70.8	30–115
2	2/15	110	30	0.62	22	65.0	46–84
	2/2	70	30	0.49	14	68.9	17–92
	15/15	51	29	0.57	10	66.2	43–79
	15/15^1^	-	-	-	10	103.6	80–123
3	2/15	136	30	0.26	20	53.6	30–64
	2/2	83	26	0.31	16	52.6	39–62
	15/15	52	17	0.33	10	63.2	48–73
	15/15^1^	-	-	-	10	79.7	74–90
4	2/15	62	25	0.40	12	50.9	33–61
	2/15^1^	-	-	-	12	72.9	62–87
	2/2	273	53	0.41	53	57.5	30–75
5	2/15	224	45	0.25	45	52.6	30–64
	2/2	44	8	0.18	9	57.9	44–72
	15/15	231	46	0.51	46	65.0	41–78

^1 ^The number of individuals in the susceptible pools was low (10 to 12 birds), hence an additional susceptible pool was created, which contained the next most susceptible 20% of birds.

Total No., total number of individuals in the F × BT combination; Resistant: number of individuals included in the resistant pool (No.), and proportion of individuals that survived to the end of test (Prop); Susceptible: number included in the susceptible pool (No.), with their mean and range of survival times in days.

### Densitometric genotyping

All pools were densitometrically genotyped for microsatellite or SNP markers as described in methods, and differences in allele frequency between resistant and susceptible pools (D-values) were calculated. In order to validate the markers that had high D-values, 35 microsatellite markers were retested across the resistant and susceptible pools of all family × BT combinations. The correlation between first and second genotyping was calculated for each marker separately across all pools. Except for one outlier with a correlation of 0.52, the individual marker correlations ranged from 0.70 to 1.0, with an average of 0.93. Including the outlier, the overall correlation between the two genotypings was 0.87. Since this correlation represents a double path (from first genotyping to actual pool content, and from actual pool content to second genotyping), this observed correlation of 0.87 is equivalent to a correlation of 0.93 between a single genotyping and actual pool content. This high correlation confirms the reliability and accuracy of the pool genotyping results as representing the content of the pools. Nevertheless, since both genotypings were done on the same pools, the high correlation does not relate to the degree to which the content of the pools represents the genotypes of the individuals making up the pool.

### Threshold PFP values for statistical significance

Table 2 shows the distribution of the comparison-wise error rates (P-values) of all four statistical tests for marker-QTL linkage: Z-test (Z), Chi-Square (CS), interval analysis (IA), and ANOVA, into bins of width 0.10 units. On the null hypothesis, the proportion of tests in each bin is expected to be 0.10. For all tests except IA, there was an excess of P-values in the lowest (0.00–0.10) bin, with the proportion of P-values in this bin ranging from 0.16 for ANOVA, to 0.25 for CS test. The estimated number of tests representing linkage to a QTL (n_1_), was 63, 62, 510, and 38 for Z, CS, IA, and ANOVA. Taken as a fraction of all tests (N), these comprised 22.9%, 22.5%, 4.0% and 13.8%, of all tests, respectively. Possible reasons for the low proportion of tests representing linkage for the IA and ANOVA tests are discussed in Methods.

**Table 2:** Distribution of P-values for ANOVA, Z-test, Chi-square (CS) and Interval analysis (IA)

Bin	AN	Z	CS	IA
0.0–0.1	0.16	0.20	0.25	0.08
0.1–0.2	0.15	0.13	0.13	0.09
0.2–0.3	0.07	0.11	0.09	0.12
0.3–0.4	0.09	0.09	0.06	0.09
0.4–0.5	0.11	0.08	0.07	0.14
0.5–0.6	0.09	0.06	0.07	0.09
0.6–0.7	0.08	0.08	0.10	0.07
0.7–0.8	0.08	0.07	0.06	0.06
0.8–0.9	0.08	0.09	0.08	0.10
0.9–1.0	0.08	0.08	0.09	0.16
N	275	275	275	12753
t_N_	237	212	213	12753
f_N_	38	63	62	510
PFP	--	0.026	0.048	0.002
o_N_	0	28	52	184
Power^1^	0.00	0.36	0.67	0.36

^1 ^Power calculated as O_N_(0.80)/f_N _to account for 20% false positives included in O_N_.

N, the total number of tests; t_N_, the estimated number of tests representing true null hypotheses; f_N _the estimated number of tests representing falsified null hypotheses (i.e., true marker-QTL linkage); PFP, the 0.20 PFP CWER P-value thresholds; o_N_, the observed number of marker tests that passed the significance threshold; Power, the estimated power of the test; ND, not done.

Based on the distribution of marker P-values for the various tests, P-values corresponding to the 0.20 PFP threshold level were 0.026, 0.048 and 0.002 for Z, CS and IA, respectively. For ANOVA, none of the marker tests reached a 0.20 PFP threshold level. Nevertheless, in all cases, P-values for ANOVA tracked those for Z very closely. Thus, the ANOVA provided strong support for the technical accuracy of the Z-tests, but did not provide information additional to that provided by the Z-tests. Hence, further results for ANOVA are not presented or discussed.

The number of significant markers for each test, and the statistical power of the test are also presented in Table 2. Power was generally low, indicating that many additional markers may have been in linkage to QTL but did not reach statistical significance. This is to be expected. As a result of the map expansion in the F6, markers "move away" from their QTL. Hence, the significance of the test drops, reducing power, even though the marker is still in linkage with the QTL. Power for CS was greater than for Z. This is probably due to the fact that CS will often be significant when Z is significant, and in addition, CS will also be significant when interactions are important, while Z will not be significant in these instances. Power for IA is also presented, but does not carry much meaning, as the proportion of tests estimated as representing linkage is very low for this test.

### QTLR

Table 3 shows P-values for Z, CS and IA for 60 markers for which Z or CS, or both were significant at the PFP = 0.20 threshold. In addition, 14 markers are listed that were part of a sequence of significant markers defining a QTLR, although they did not reach significance for Z or CS. Of the 60 markers with significant tests, 10 were not included among the QTLR, because they were significant for a single test only, or were not part of a sequence of two or more markers each with at least one significant test. Of the remaining 50 markers, 36 were significant for CS with strong support from IA. Correspondence between CS and IA is particularly striking in the instances where both differed widely from Z. Eight markers were significant for CS, but were not supported by IA, and hence were initially not considered as significant for purposes of QTLR definition. However five markers (on chromosomes 9 and 15) that were excluded initially as not meeting criteria, were included among the final list QTLR, because they corresponded to QTLR previously mapped by [18]. This returned four markers that were significant for CS, but not supported by IA. In all, 52 markers were considered as defining QTLR. Of these, 27 were significant for CS only, while 25 were significant for Z, of which 11 were significant for Z only and 14 were significant for Z and CS. Joint significance of Z and CS basically means that CS is supporting Z, since large uni-directional D values will generate significance for both tests. Since power for CS is less than for Z, it is expected that some markers will be significant for Z but not for CS. Thus, about half of the significant markers represented significant main effects, while the other half represented significant interaction effects without significant main effects.

**Table 3:** Markers significant for Z-test and/or Chi-square (CS): P-values for Z-test, CS and interval analysis (IA)

Marker	Chromosome	Pos	Z	CS	IA	Type
MCW0106	1	94	0.60	0.00	0.02	C
						
ADL1245	1	245	0.00	0.00^§^	0.99	Z
ADL1248	1	248	-0.02	0.63	0.93	
						
LEI0217	1	299	-0.03	0.00	0.14	ZC
						
HYL0238	2	45	-0.48	0.04	0.10	C
*ADL0270	2	46	-0.35	0.14	0.11	
ADL0270r	2	46	-0.25	0.01	0.11	C
						
*HYL0247*	2	54	-0.98	0.01	0.11	
						
MCW0063	2	119	0.01	0.67	0.62	Z
MCW0239	2	126	0.00	0.28	0.38	Z
						
*ADL0157r*	2	245	0.09	0.01	0.46	C
						
MCW0257	2	272	0.18	0.00	0.02	C
MCW0257r	2	272	0.15	0.00	0.02	C
*MCW0288	2	277	0.61	0.19	0.19	
MCW0288r	2	277	0.40	0.01	0.19	C
						
MCW0051	2	358	0.02	0.03	0.02	ZC
MCW0051r	2	358	0.04	0.00	0.02	C
ADL2361	2	361	0.01	0.00	0.00	ZC
*MCW0245	2	364	0.11	0.10	0.03	C
MCW0245r	2	364	0.01	0.00	0.03	ZC
						
*ADL2374	2	374	0.04	0.82	0.61	
MCW0282	2	378	0.01	0.29	0.17	Z
MCW0282r	2	378	0.00	0.09	0.17	Z
						
CPPP	3	292	0.00	0.05	0.22	ZC
						
*MCW0005*	4	75	0.26	0.01	0.30	
						
*UMA4027	4	137	-0.12	0.26	0.18	
UMA4027r	4	137	-0.07	0.03	0.18	C
*HYL0437	4	138	-0.14	0.09	0.17	
HYL0449	4	144	-0.43	0.01	0.10	C
						
*HYL0515*	5	31	0.27	0.03^§^	1.00	
						
*ADL0292	5	83	-0.04	0.18	0.20	
*ROS0052	5	90	-0.05	0.63	0.55	
MCW0078	5	93	0.00	0.03	0.11	Z
*ADL0312	5	95	-0.06	0.52	0.39	
ADL0023	5	96	-0.02	0.29	0.34	Z
						
MCW0081	5	152	-0.02	0.00	0.00	ZC
MCW0081r	5	152	-0.01	0.00	0.00	ZC
ADL0166	5	162	0.03	0.00	0.00	ZC
ADL0166r	5	162	0.02	0.00	0.00	ZC
						
ADL0298	5	198	0.02	0.00	0.11	ZC
ADL0298r	5	198	0.37	0.00	0.11	C
						
*MCW0305*	8	15	-0.70	0.00^§^	0.97	
						
HYL8003	8	40	0.00	0.00	0.47	ZC
						
*HYL8033*	8	59	-0.02	0.42	0.53	*Z*
						
*ROS0307*	8	96	0.42	0.00^§^	0.99	
						
LEI0028r^1^	9	51	0.50	0.03^§^	0.65	
LMU0006^1^	9	51	-0.97	0.00^§^	0.65	
*HYL0928^1^	9	55	-0.22	0.33	0.92	
LEI0197^1^	9	56	0.14	0.03^§^	0.91	
LEI0197r^1^	9	56	0.00	0.00^§^	0.91	Z
						
ADL0259	9	122	-0.01	0.00	0.00	ZC
						
*MCW0244*	13	16	0.04	0.03	0.37	C
						
MCW0052^1^	15	26	0.10	0.01^§^	0.63	
						
HYL1808	18	23	0.00	0.00	0.01	ZC
MCW0217	18	24	-0.07	0.03	0.01	C
MCW0217r	18	24	-0.06	0.02	0.01	C
*HYL1809	18	26	-0.04	0.07	0.03	
HYL1816	18	32	-0.09	0.00	0.22	C
						
*MCW0209*	26	36	-0.15	0.03	0.28	
						
*HYL2612	26	66	0.15	0.10	0.09	
LEI0074	26	67	0.55	0.00	0.08	C
LEI0074r	26	67	0.26	0.04	0.08	C
ADL2668	26	68	0.64	0.00	0.01	C
*ADL2669	26	68	0.92	0.82	0.01	
*ADL2669r	26	68	0.24	0.00	0.01	C
						
*ROS0309	Z	36	0.12	0.75	0.86	
MCW0055	Z	37	0.00	0.01§	0.63	Z
MCW0258	Z	42	0.02	0.24	0.81	Z
MCW0331	Z	43	0.02	0.11	0.86	Z
*ROS0301	Z	49	0.09	0.92	1.00	
						
*ADL0273*	*Z*	52	0.33	0.01^§^	0.53	
MCW0241	Z	74	0.01	0.00^§^	0.50	Z
						
MCW0227	Z	87	-0.23	0.03	0.24	C
LEI0121	Z	97	0.50	0.01	0.26	C
LPL	Z	103	0.59	0.00	0.03	C

^1^These markers were significant for CS, but were not supported by IA. They are included in the list of QTLR, because they corresponded to QTLR previously mapped by [18].

PFP thresholds: Z, 0.028; CS, 0.048; *, Non-significant but part of a linked series of significant markers defining a QTLR; Italics, markers not included in final count of QTLR because significant only for a single test and marker; §, CS not supported by IA; Type, tests for which marker is significant; r at the end of a marker name represents a repeat test of the same marker; Spaces between rows delineate the individual QTLR.

Based on Table 3, QTLR and non-Q regions, as defined in Methods, are listed in Table 4, which shows the total number of markers in each region (counting also non-significant markers incorporated within a QTLR) and the location of the markers defining the start and end points of each region. For QTLR, the statistical tests for which the region is significant, the estimated allele substitution effect on survival time, and overlap with regions of significance in Heifetz et al. [18] and Yonash et al. [21] are also shown. A total of 21 QTLR were identified. Of these, 8 were significant for CS only, while 13 were significant for Z either alone (6) or for Z as well as CS (7). For six of the regions, QTL location and confidence interval, as determined by IA, are also shown in the table.

**Table 4:** Chromosomal regions that contain QTL (QTLR) and that do not contain QTL (non-Q)

Chr	Reg.	Type	M	Test	Location (cM)	Effect (days)	BC/Yonash
1	I	Non-Q	2		33–79		
	II	QTLR	1	ZC	94; *95 (85–101)*	1.62	
	III	Non-Q	10		122–243		
	IV	QTLR	2	Z	245–248	0.39	
	V	Non-Q	15		259–523		
							
2	I	Non-Q	1		44		
	II	QTLR	3	C	45–46; 45	-2.49	Y (34–60)
	III	Non-Q	13		47–113		
	IV	QTLR	2	Z	119–126	7.81	BC 5.74^1 ^(82–112)
	V	Non-Q	17		155–261		
	VI	QTLR	4	C	272–277: *273 (269–275)*	2.88	
	VII	Non-Q	5		278–326		
	VIII	QTLR	5	ZC	358–364; *361 (343–368)*	6.53	
	IX	QTLR	3	Z	374–378	7.18	
	X	Non-Q	4		389–400		
							
3	I	Non-Q	16		9–291		
	II	QTLR	1	ZC	292	-8.00	
	III	Non-Q	1		295		
							
4	I	Non-Q	11		50–125		
	II	QTLR	4	C	137–144	-4.69	Y (106–124)
	III	Non-Q	8		148–188		
							
5	I	Non-Q	9		8–34		
	II	QTLR	5	Z	83–96	-5.46	
	III	Non-Q	2		106–122		
	IV	QTLR	4	Z	152–162: *162 (160–165)*	-1.15	BC -3.56^1^(140–153)
	V	Non-Q	2		168–198		
	VI	QTLR	2	C	198	4.74	
							
6	I	Non-Q	4		41–141		
							
7	I	Non-Q	5		0–135		
							
8	I	Non-Q	8		8–39		
	II	QTLR	1	ZC	40	11.25	BC 9.78 (43–56) Y (30–38)
	III	Non-Q	9		41–109		
							
9	I	Non-Q	9		43–50		
	II	QTLR	5	C	51–56	2.07	BC 4.52 (48–56)
	III	Non-Q	2		57–71		
	IV	QTLR	1	ZC	122: *124 (101–134)*	-7.00	
							
11	I	Non-Q	2		0–68		
							
13	I	Non-Q	7		16–44		
							
15	I	Non-Q	11		1–26		
	II	QTLR	1	ZC	26	4.69	BC 6.40 (3–39)
	III	Non-Q	1		39		
							
18	I	Non-Q	2		7–20		
	II	QTLR	5	ZC	23–32: *23 (22–28)*	-5.53	
							
26	I	Non-Q	3		33–38		
	II	QTLR	6	C	66–68: 64	2.15	
							
Z	I	QTLR	6	Z	36–52	5.87	BC 9.13^1^(0–49)
	II	QTLR	1	C	74	4.04	BC 5.95^1^(52–74)
	III	QTLR	3	C	87–103	0.30	BC 7.77^1 ^(103–115)
	IV	Non-Q	1		115		

^1 ^Also supported by McElroy et al., (2006)

Chr., chromosome; Reg., region; M, number of markers in the region; Test, Z, significant by Z-test; C, significant by Chi-square and supported by IA; Location, cM of the markers flanking the region, in italics following semi-colon location and (confidence interval) by IA; Effect, allele substitution effect of the region; BC, allele substitution effect and location in Heifetz et al. (2007); Y, region of significance in Yonash et al. (1999).

Eight of the QTLR corresponded to QTLR that were previously identified by Heifetz et al. [18] in the reciprocal BC populations generated from these lines. As noted above, however, two of these (QTLR 9-II and 15-II) were of borderline significance in the F6 and were included among the F6 QTLR because of support from the Heifetz et al. [18] study. In addition, three of the QTLR corresponded to significant QTL reported by Yonash et al. [21], of which one was also reported in the Heifetz et al. [18] study. Thus 11 out of 22 QTLR identified in the F6 were supported by other studies of these lines or of other Leghorn layer lines.

The total absolute allele substitution effect summed across all 21 QTLR was 95.84 days, with a mean absolute allele substitution effect of 4.56 days. This compares well with the mean absolute allele substitution effect of 5.53 days for the QTLR identified in the Heifetz et al [18] study when based on combined data of the two BCs. Of the 21 QTLR, 14 presented positive effects, indicating that the resistant allele originated from Line 2 (the more resistant line), and 7 presented negative effects, indicating that the resistant allele originated from Line 1 (the less resistant line). This corresponds to the results of the BC analysis [18], in which case also resistance alleles originating from Line 1 were uncovered. The total summed effect for survival time, across all 21 loci including algebraic sign, was 27.20 days in favor of Line 2. Although not directly comparable, this is in accord with the observed difference of 21.0% in proportion of survivors in favor of Line 2, found in the present study.

When some of the same QTL are uncovered in independent genome scans, this supports the validity of these QTL. However, since independent scans will have some QTL in common and some that differ, it is always possible that at least some of the shared QTL represent chance Type I errors that happened to occur in the same chromosomal region. The likelihood of this decreases when the common QTL share specific qualities, other than their quantitative effect. For example, in a previous study of QTL affecting milk yield and protein percent in Brown Swiss dairy cattle [22], it was found that QTL identified in the Brown Swiss breed shared specificity (for milk yield or for protein percent, or both) with those identified in the Holstein breed. This was taken as strong support for the reality of these QTL. Similarly, in the present study, the identified QTL have specificity in terms of direction of effect (whether the allele derived from the more resistant line had a positive of negative effect on resistance) and magnitude of effect. It is striking, therefore, that of the 8 QTLR that were found in the present study and also in the Heifetz et al. [18] study, the same 7 had positive effects in both studies, and the same one had a negative effect in both studies. The likelihood of either of these events is low, although within the realm of chance. Furthermore, the correlation between estimated signed allele effects of the common QTLR in the F6 and BC was 0.67 (P < 0.03); the correlation increased to 0.84 (P < 0.001) if an exceptional value (for QTLR Z-III) is excluded. Thus, the nearly significant correspondence of algebraic sign for the common QTLR of the two studies, and the significant correlation of their estimated effects strongly supports their underlying reality. The preponderance of positive effects among the QTLR that were common to the two studies, suggest that these are The QTLR that contributed to the superior resistance of Line 2.

The mean minimum and maximum width of the QTLR identified in this study was 4.73 and 14.93 cM respectively; if QTLR defined by a single marker are excluded, the minimum extent becomes 7.42 cM. The mean extent of a QTLR, as defined by IA was 17.0 cM. Comparable values calculated for the QTLR defined in the Heifetz et al. [18] BC study (calculated from Table 3 of [18]) were a minimum extent of 13.4 cM (20.3 cM when excluding QTLR defined by a single marker); a maximum extent of 34.1 cM, and a mean extent from IA of 24.6 cM. The mean extent for the F6 QTLR, defined by all four measures, was 11.02 cM; the corresponding value for the BC QTLR was 23.00. Thus, QTL map resolution appears to have been reduced by half in the F6 as compared to the BC populations. This corresponds almost exactly to the calculated degree of map expansion in this population based on unpublished data (J.E. Fulton and E. Lipkin pers. comm.).

## Discussion

MHC type B15/B15 was clearly more resistant than B2/B2 and B2/B15, but the resistance appeared to be fully recessive, as the heterozygote was virtually identical to the B2/B2 homozygote. Similar results were obtained in the reciprocal backcross study of these founder lines [18]. This was somewhat unexpected, as a number of studies comparing the influence of various MHC haplotypes on MD in different strains of chickens found that the B2 haplotype is frequently associated with greater resistance [23,24]. These data are, however consistent with others that suggest that some genes may interact to complement the MHC haplotype influence [25].

The QTL results of this study accord well with those of the previous reciprocal backcross study of these lines [18]. In the BC case, a total of 15 QTLR were identified. Of these, 3 QTLR were significant for CS only, 12 were significant for ANOVA or Z, of which 8 were also significant for CS. Three-fifths of the QTLR identified in the previous study were also identified in the present study, and the effects of the common QTLR were identical in sign, and highly correlated in magnitude.

Considering the three main MD resistance QTL-mapping studies, the present F6 and the BC [18] experiments between them identified all four of the significant Yonash et al. [21] QTL; 8 QTL were common to the F6 and BC [18] experiments; while 13 and 7 were uniquely identified by the F6 and BC [18] experiments, respectively. Thus, among them these experiments uncovered a total of 28 QTL affecting MD resistance. Lack of full correspondence between the present F6 and previous BC [18] experiments, which were carried out in populations derived from the same founder lines, can be attributed to the partial power of the experiments. Assuming a total of 28 QTL, power of the F6 experiment would be 0.77 (21 out of 28) and that of the BC experiments would be 0.54 (15 out of 28). These power estimates seem reasonable considering the size and design of the experiments and magnitude of the QTL effects uncovered, and the difference between the power estimates for the F6 and BC experiments is far from statistical significance. For experiments of comparable size and QTL of comparable effects, F2 and reciprocal backcross designs should provide equivalent statistical power [26]. Thus the observed difference in power of the two experiments is best attributed to sampling variation.

Overall, Line 2 contributed about twice as many resistance alleles as Line 1, as would be expected from the relative resistance of the two lines. Curiously, in the present study, the pure line controls of Line 1 and Line 2 displayed much higher resistance (28.6 and 7.6% mortality to end of test, respectively), compared to the F6 (52.7% mortality to end of test). Thus, interactions of the background genome appear to have major effects on the expression of resistance [27]. The fact that the cross of the two lines was more susceptible than each of the individual lines is consistent with other indications that the QTL alleles that confer resistance in these lines are recessive, so that the cross shows negative heterosis for resistance. This stands in some contrast to the results of Stone [28], who conducted a number of crosses between ADOL resistant and susceptible lines and concluded that MD resistance was dominant, and of Yonash et al. [21], who found that at a majority of their identified QTL, the resistant alleles were dominant. The results are, however, consistent with the Heifetz et al. [18] study, in that QTL mapping in the reciprocal backcross populations developed from these founder lines identified different QTL, as would be expected if QTL for resistance are recessive. The hypothesis that resistance alleles are recessive also predicts that the F6 should identify QTL that came to expression in both of the reciprocal BC populations. In this regard, eight of the QTL identified in the BC populations corresponded to QTL identified in the F6. Of these, one was specific to BC1, three were specific to BC2, and four were found in both BC's. Seven of the QTL mapped in the BC, however, did not have corresponding QTLR in the F6. The simplest explanation for these negative results may be the incomplete power of the two experiments, which appreciably reduces the likelihood that the same QTL will be found in the two experiments.

Three of the QTLR identified in the F6 were also identified in the Yonash et al., [21] study. This is particularly noteworthy, since the challenge strain in the Yonash et al. [21] study was JM/102W, which is a virulent (v) MDV strain, but less pathogenic than the vv+ MDV strain 648A used in the present study. Thus, this lends some support to the widely held assumption that QTL conferring resistance to one MDV strain will confer resistance to another as well, at least in White Leghorns where genetic diversity is limited.

Considering QTL map locations in the BC as compared to the F6, there is a clear tendency for distinctly narrower QTLR in the F6 than in the BC; average QTLR extent in the F6 was just about half that in the BC, as anticipated from the observed map expansion in the F6. Additional genotyping at higher marker density across the QTLR is clearly warranted to obtain full benefit of the F6 map expansion.

Counting all resistance QTL uncovered in the two BC populations by Heifetz et al. [18] and in the F6 in the present study, gives a total of 28 QTL; eight common to both series of crosses, 13 uncovered only in the F6, and 7 uncovered only in the BC populations. Of the 8 common QTLR, 5 were significant by Z or by Z and CS, indicating important main effects. These results should provide a strong platform for comparative positional cloning, after confirmation of the associations by individual genotyping of the pools. Comparative functional genomics based on the complete chicken genome sequence could be used to identify candidate genes in the identified chromosomal regions. As a preliminary exercise, we have searched Build 2 of the chicken genome across 5 cM centered at each of three regions which had narrow widths in the present study, and corresponded to regions of significance in [18] or [21], namely: QTLR 2-II (supported by [21]), centered at ADL0270 at about 9.7 Mb; QTLR 8-II (supported by [18] and [21]), centered at HYL08003 at about 18.5 Mb; and QTLR 9-II (supported by [18]), centered at LEI0197 at about 13.0 Mb. The most likely candidate gene in QTLR 2-II is PTPRN2 (protein tyrosine phosphatase, receptor type, N polypeptide 2) at 8.7 Mb (1 Mb downstream of ADL0270). PTP family members are signalling molecules that regulate many cellular processes, including cell growth, and have been implicated in oncogenic transformation. QTLR 8-II contains two interesting candidate genes: CD97 antigen, at 19.2 Mb (~0.7 Mb downstream of HYL08003) and PIGK (phosphatidylinositol glycan, class K), at 19.9 Mb (~1.4 Mb upstream of HYL08003). CD97 antigen is a receptor involved in cell adhesion and signalling, that is present on the surface of most activated leukocytes. MDV is thought to infect and transform only activated CD4+ T cells. Consequently, cell adhesion might assist the virus transmission from one infected cell to another, as MDV is highly cell associated. PIGK is a subunit of the GPI transamidase complex that catalyzes the attachment of GPI (glycosylphosphatidulinositol) to proteins. GPI is a membrane anchor for cell surface proteins. As such it provides for rapid protein release in response to a stimulus, since the protein bound by the GPI anchor can be immediately released without a requirement for RNA or protein synthesis. In this context it is relevant that SCA2 (stem cell antigen 2) has a GPI anchor, and is one of the most strongly documented MD resistance genes [9]. For QTLR 9-II, using LEI0197 (at 13.0 Mb) as the reference point, we were unable to find any attractive candidate genes. This may in part be because the biology of MD resistance is not well defined. A further contributing factor is the fact that much of the chicken gene annotation is inferred by electronic annotation and not by experimental evidence. Consequently, one can speculate about almost any gene being involved in viral replication and spread, or cellular transformation, especially as the biomedical literature is slanted heavily towards cancer.

These and other candidate genes can be screened further by examining their mRNA expression pattern under MDV challenge, using existing data banks. However, they could also readily be examined for linkage disequilibrium with MD resistance by constructing appropriate resource populations using the existing data and sample banks accumulated at Hy-Line through the routine MD challenge and testing component of their regular commercial breeding program. Such association tests could also be implemented by selective DNA pooling, perhaps using the new fractioned pool designs [29] to increase power and accuracy.

## Conclusion

If the resistance alleles are recessive and at low to moderate allele frequencies, this would explain the slow response to selection for increased resistance, and enhances the need for mapping in order to increase the effectiveness of selection on these QTL within the pure lines. Because most chickens used for commercial egg or meat production are crosses between 2 to 4 lines, it will be important that the recessive resistance alleles are present in all pure lines, such that the cross will be homozygous for the resistance QTL. For lines that lack the resistance alleles, which would need to be determined as a first step, it might be most effective to introgress resistance alleles from one line to another. Achieving this, without losing the heterosis for production traits that characterizes the cross of these commercial lines, could only be achieved through marker or preferably gene assisted introgression focused on the limited genome regions carrying the resistance alleles.

## Methods

### Resource population

#### Stocks

The experiment was carried out using facilities and two commercial White Leghorn lines (henceforth, Line 1 and Line 2) of Hy-Line International (henceforth, Hy-Line). Both lines were partially inbred and had been subjected to selection for resistance to MD, and both were relatively resistant when compared to field strains of poultry. However, under the same challenge protocol as the present study, Line 2 was distinctly more resistant than Line 1. Further details of these lines are in Heifetz et al. [18].

#### Experimental populations

**Figure 1:**
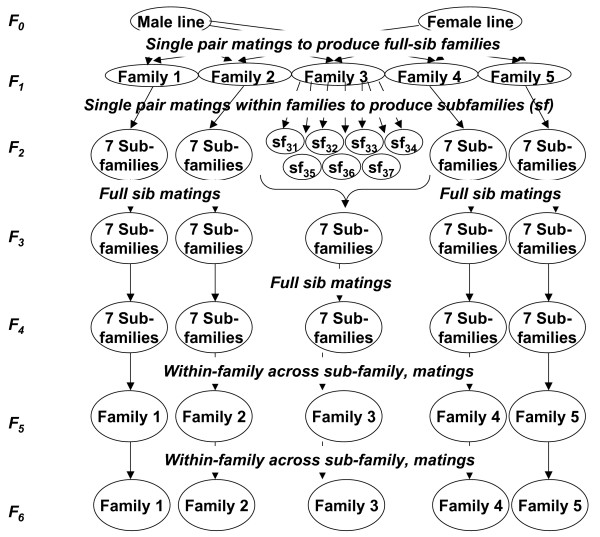
**The experiment design**.

In order to provide replication and some indication of QTL segregation within the two lines, the F6 FSIL was produced in five independent replicates, termed "FSIL-families", as follows (Figure 1): Five Line 1 males were each pair-mated with a single Line 2 female, to produce an F1 generation consisting of five large and independent full-sib F1 families. Each of these F1 families served as the founder of one of the replicate FSIL-families. Within each of these five F1 founder families, 7–10 males were randomly mated to two females each, to produce seven F2 subfamilies within each of the five FSIL-families. Each of the F2 subfamilies within each of the five FSIL-families was then continued by crossing full-brother and sister within the subfamily for another two consecutive generations, creating at the F4 generation five replicate FSIL-families, each consisting of 7 partially inbred subfamilies. This was done to maintain genetic diversity within each family, while still accumulating recombination events. At the F4 generation, males from each of the seven subfamilies within each FSIL-family were crossed to females of other subfamilies within their own FSIL-family (but not with their own full-sisters, or with females of the other FSIL-families). In this way, the five separate F5 FSIL-families were reconstituted. In the F5 generation, a total of about 30 males and 120 females per FSIL-family were chosen and mated at random to produce the five independent F6 replicate FSIL-families, which constituted the mapping population. The F6 birds were produced in two hatches, with a total of 862 and 753 female chicks for Hatches 1 and 2, respectively. The total number of female F6 birds within each of the five FSIL-families with full data (survival time, MD diagnosis) ranged from 98 to 248.

### MD challenge test

Day-old female F6 chicks from the two hatches were vaccinated with 500 plaque forming units (pfu) of bivalent HVT/SB-1 vaccine (Merial Select, Gainesville, GA, 30503) and then housed in cages. A total of 200 pure line chicks from each of the two parental lines were included as controls for each hatch of the F6 (800 chicks total). At 7 days of age, the chicks were inoculated subcutaneously with 500 pfu of the very virulent plus (vv+) strain (648A) of MDV [30]. Age at mortality was recorded on all chicks, as an indicator of MD resistance, until 116 and 123 days of age for Hatches 1 and 2 respectively, at which time remaining birds were terminated. Under normal circumstances, mortality in these lines is virtually zero (0.5%) from 2 to 18 weeks of age. Under challenge, MD mortality does not begin until four weeks of age at the earliest. After this age, however, virtually all mortality in the challenged birds is due to MD. Therefore, any chick that died after 21 days was considered to have died from MD, without positive diagnosis by necroscopy. At the end of the experiment at 18 weeks (126 days, just prior to entry into lay), the birds were visually examined and any birds that were blind, lame or completely paralyzed were identified as having MD. However, birds with mild symptoms or birds that had recovered, were not recognized as such, and hence were classed as survivors. Thus, the resistant pools, which were made up of survivors, may also have included some proportion of susceptible birds with MD tumours. Target mortality for the challenge test averages about 50%, but in any particular hatch this varies considerably, from 30 to 70%. The challenge test and diagnosis procedure has been shown to be repeatable and to result in data with substantial heritability for sire progeny averages based on 30 daughters (pers. comm., Neil O'Sullivan, Hy-Line Int.).

### DNA extraction and pool construction

In order to reduce the number of genotypings while limiting loss of power, the selective DNA pooling method [31,32] was used. This method has proven very accurate in our laboratory [33]. Ten and six days post infection for Hatches 1 and 2, respectively, blood was collected from the jugular vein in syringes containing EDTA with 22 gauge needles. DNA was prepared using salt and ethanol precipitation. The OD 260/280 ratios were determined and each sample was diluted to approximately 50 ng/μl DNA concentration. DNA content was retested and further diluted to 25 ng/μl. Pools of DNA were made by combining equal volumes of the 25 ng/μl samples from each bird in the pool.

Mortality rate was very similar for the two hatches (56.2 and 57.2%, respectively) and was also consistent across hatches for the five families considered separately (data not shown). For each F6 family, therefore, chicks from the two hatches were combined when forming pools. There were three blood type (BT) groups in each F6 family: B2/B2, B2/B15, and B15/B15. To account for the well known effects of MHC blood type on resistance to MD [34-36], birds were pooled within B blood groups. For selective DNA pooling, progeny within each family × BT combination were ranked by age at mortality and the top and bottom 20% from each combination were chosen for the pools (Table 1). This gave 30 pools in total (5 families × 3 BT × 2 tails (high and low)). However, the Family 4 × B15/B15 combination had only 5 birds in total, which reduced the total number of pools to 28. The remaining pools ranged in size from 9 to 53 individuals. For most of the susceptible pools, the cut off survival age to be included in the pool varied from 62 to 79 days. However, two pools (Family 1 × B15/B15, and Family 2 × B2/B2) included birds surviving up to 115 and 92 days, respectively. In family × BT combinations with small numbers, the number of individuals in the susceptible pools was low (10 to 12 birds), hence an additional susceptible pool (Type II pool) was created, which contained the next most susceptible 20% of birds (cut off points demarcating the two pools can readily be inferred from the range column of Table 1). There were four susceptible pools of this type. With respect to the resistant pools, in most family × BT combinations the proportion of surviving (i.e., resistant) birds exceeded 20%, since overall survival to end of test was 56%. In this case, birds for the resistant pools were chosen so as to ensure that each hatch was equally represented. In all, then, there were a total of 32 pools: 14 resistant pools and 18 susceptible pools (14 type I and 4 type II).

### Genotypic information

#### Markers

Microsatellite markers were chosen that had alleles differentiating the two lines. Genotyping of pools was performed in two rounds. The first round included 182 markers, chosen to provide representative genome coverage. Based on the first round of genotyping, 35 markers that gave a suggestive result were genotyped again on the same pools. These results were treated in the final analysis as though they were new markers at the same location. In addition, in 10 regions that showed results of special interest in the first round, 50 new markers were added, among them 35 microsatellites and 15 SNPs. Thus, in total the pools were genotyped for 232 markers: 15 SNPs and 217 microsatellites. However, since 35 markers were genotyped twice, and the replicates were treated as new markers, all told there were 267 single-marker statistical tests. The chromosomal regions spanned by the genotyped markers summed to a total of 2477 cM. Adding 10 cM upstream and downstream of the most distal and proximal markers for each of the 15 scanned chromosomes would add another 300 cM, giving a total of about 2800 cM, or about 70% of the chicken genome. The average distance between markers on the same chromosome was ~17 cM, with a maximum interval of 107 cM on chromosomes 3.

#### Marker position

The interval analysis method used for QTL mapping requires recombination rates between markers. To obtain these rates, markers were positioned on a linkage map based on the consensus 2000 map [37]. When there was a discrepancy between the consensus map and the published Build 1 genome sequence [38], the order of the markers was taken according to the sequence and markers were positioned as described in Heifetz et al. [18].

Genetic distances between markers in cM, as given in the public marker maps, are based on data obtained in F2 or BC populations, and hence represent effects of a single round of recombination. For AIL, infinitesimal map distances expand according to the expression d_t _= 1/2 td, where d_t _is map distance in the F(t) generation, and d is the map distance in the F2 generation [15]. According to this expression, for an F6 population, the overall expansion factor is three-fold. For interval analysis, map distances calculated in this manner were transformed to recombination rate (r) values using the Haldane map function. The three-fold map expansion applies to AIL with random mating. Here, the lines were propagated by brother-sister matings to generate the F3 and F4 generations, which created some level of inbreeding and may have reduced the actual expansion of the map. Indeed, studies under way indicate that the realized expansion was two-, rather than three-fold. Therefore, the interval analysis was done using marker maps calculated with an expansion factor of 2, 3 and 4 to check the sensitivity of the results to this parameter.

### Genotyping methods

Genotyping for the B group was done by standard serotyping using B2 and B15 specific reagents. For the microsatellite markers, allele frequency differences between high and low pools were estimated as described by Heifetz et al. [18]. For eight of the SNP markers, relative allele frequencies were determined by pyrosequencing. In brief, mini-sequencing primers were designed by the Pyrosequencing Assay Design Software (version 1.0) (Biotage, Uppsala, Sweden). The forward, biotinylated reverse, and sequencing primers were synthesized by Operon (Huntsville, AL). Fragments were amplified in a total volume of 40 μl by PCR as described by Liu and Cheng [39]. The biotin-ssDNA was isolated by binding PCR amplicons to streptavidin sepharose (Amersham Biosciences, Uppsala, Sweden) followed by denaturation with 0.2 M NaOH. The mini-sequencing primer was annealed to the ssDNA, and the sequencing reactions were initiated using a nucleotide dispensation order based on the sequence. The PSQ96MA analysis program (Biotage, Uppsala, Sweden) calculated a percentage for each base at the SNP using the surrounding peaks as reference values. Controls included absence of template and known homozygous and heterozygous individuals. Using the same procedures but calling genotypes instead of allele frequencies, the remaining seven SNP markers were genotyped individually on all individuals that constituted the pools. For these markers the frequency in each pool was the actual frequency of the alleles in the pool and was treated in the analyses as a pool frequency estimate.

### Statistical methods

#### Frequency estimates and D-values

Following Lipkin et al. [32], the frequencies of alleles at each microsatellite marker estimated from pools were corrected for differential amplification and for the shadow bands that are inherent to microsatellite markers. The basic datum that was used to identify markers that are associated with QTL for MD was the difference (D_ijk_) in allele frequency of the i^th ^marker between the resistant and susceptible pools of the j^th ^BT × k^th ^family combination: D_ijk _= dF_ijk1 _- dF_ijk2_, where dF_ijk1 _and dF_ijk2 _are the densitometric estimates of the frequency of the marker allele derived from Line 2 in the resistant and susceptible pools, respectively.

A total of 18 D_ijk _were calculated for each marker. These included 14 D_ijk _based on the resistant pools and the Type I susceptible pools; and an additional four D_ijk _based on the four Type II susceptible pools and their corresponding resistant pools (which are the same as for the corresponding Type I pools). Although the four Type II D_ijk _are not independent of their corresponding Type I values (since they are from the same test population and were contrasted to the same resistant pool), they were considered as being independent in the analyses which follow.

The D_ijk _were evaluated for significance using a variety of statistical tests that were calculated across all 18 blood type × family (BF_jk_) combinations within each marker, namely: Z-test, Chi-square (CS), interval analysis (IA), ANOVA, and nonparametric Sign Test. The Z, CS and Sign tests were carried out using Excel; ANOVA was implemented using the Fit Model in JMP 5.1.2 Statistical package (1989–2004 SAS Inst. Inc.); IA was implemented by EH using programs provided by JW. Further detailed expressions and descriptions of the various tests are given in Heifetz et al., [18] and will not be repeated here. Each of these tests explored a somewhat different aspect of the data. In particular, the Z-test evaluates the main effect of a marker allele on D_ijk _across all BT × family (BF) combinations. Thus, the Z-test is sensitive to main effects and provides an estimate of the direction of the effect of specific alleles, but is insensitive to marker-BT-family interaction effects. The Chi-square test analyzes D_ijk _within each BF combination, allowing for different directions of effects and is, therefore, less sensitive to main effects than the Z-test but more sensitive to interaction effects. The Z- and Chi-square tests are both based on analysis of single markers. To take into account the additional information present in adjacent markers, D_ijk _for all markers on a chromosome were analyzed jointly using a likelihood-based interval mapping (IA) method [40], as implemented by Heifetz et al. [18] for a backcross but with additional adjustments for autosomal and Z chromosomes in an F6 population. By analyzing each BF combination as a separate family, the IA shares with Chi-square its sensitivity to interaction effects but is also less powerful than the Z-test to detect main effects. These three tests are based on the D_ijk _divided by their standard errors (SE). Two additional tests: a three-way ANOVA (marker × blood type × family) and a nonparametric sign test were also used. These, although based on the same D_ijk_, each use a different basis to test significance, in this way providing an additional control to the statistical calculations. Both ANOVA and the Sign Test share with the Z-test its sensitivity to main effects and insensitivity to interaction effects. The Sign Test was used primarily as a check on the Z-test and ANOVA, and proved effective in indicating technical errors in the analysis, but will not be presented or discussed in detail. Mean Square error in the ANOVA was estimated from the pooled interaction effects, rather than from replicated markers. Hence, considering that significant interaction effects were present in this population, the MS error term would have been subject to upward bias, which increases P-values for this test, decreasing power relative to the Z-test.

#### Accounting for multiple tests

To take into account the multiple test situation while retaining power, a 20% "proportion of false positive (PFP)" threshold was used to determine the critical comparisonwise error rate (CWER) or P-value for declaring marker-QTL linkage [41], where PFP for the i^th ^test is calculated as:

PFP_i _= (P_i_t_N_)/R_i_

P_i _is the P-value of the i^th ^test, when the N tests are ranked by their P-values from lowest to highest,

R_i _is the rank number of the i^th ^test, and

t_N _is the number of tests for which the null hypothesis is true.

Estimation of t_N _was by the Nettleton et al. [42] algorithm for ANOVA, Z and CS (see also [18]).

Given an estimate of t_N_, the number of tests representing falsified null hypotheses, f_N _(i.e., tests representing true marker-QTL linkage), can then be estimated as f_N _= N - t_N_, and effective power, as o_N_/f_N _where o_N _is the number of tests that are significant according to the designated significance level.

For the IA test, the simplified algorithm of [43] was used to estimate t_N_, which works well when t_N _is almost equal to N. The PFP calculation was done using all IA tests that were conducted on a chromosome at 1 cM intervals on the expanded map, as in the range of CWER values > 0.001 there was a fairly smooth and monotonic relationship between rank number and PFP (see also figure 2 of [44]). On this basis, PFP calculation was done using all IA tests that were conducted on a chromosome at 1 cM intervals on the expanded map.

### Effect of added markers and map expansion on PFP threshold levels

As noted above, genotyping was performed in two rounds. Markers used in the first round were chosen to provide good genome coverage, but without relation to previously identified genes or QTLR affecting MD resistance. However, the 85 markers used in the second round were specifically targeted to "suggestive" regions uncovered in the first round. These were either repeat genotypings of the suggestive markers themselves (35 markers) or additional markers targeted to the suggestive regions (50 markers). Thus, the actual proportion of positives can be expected to be greater among the second round markers than among the first round markers. Combining the two sets of markers in a single PFP analysis, as was done in the present study, will increase the actual proportion of positives among all markers and, hence, can be expected to render the PFP thresholds somewhat less stringent relative to those appropriate to the first set of markers, but more stringent relative to those appropriate to the second set of markers. This consideration will be more important for the single marker tests (ANOVA, Z, CS) than for IA, since IA deals with chromosomal regions and the number of markers in a significant region is not material.

With respect to the effect of map expansion on PFP thresholds, for Z, ANOVA, and CS the effect of map expansion should be to increase average distance between markers and QTL, and hence increase P-values for all markers except those that are very close to the QTL. Thus, there should be a general increase in P-values. This will result in more stringent PFP thresholds for the F6 as compared to the same markers in the F2 or BC, and a consequent reduction in power as compared to the BC. As will be demonstrated in results, loss of power was especially severe for ANOVA and IA. For ANOVA, loss in power due to map expansion added to the loss of power due to use of the interaction MS as error term. For IA, in addition to the reduced P-values all along the genome, there will also be a massive increase in the number of non-significant test points (after taking three-fold map expansion into account, the IA analysis included 12,753 individual points). Since the number of QTL is fixed, the ratio of QTL to test-points drops precipitously, with the vast majority of test points representing true null hypotheses. This will necessarily increase stringency of the threshold for a given PFP, since total number of test points enters PFP calculation in the numerator. For this reason, we did not relate to the actual PFP thresholds for ANOVA or IA, but to the absolute P-values, and considered ANOVA and IA as supporting significant Z-test and CS tests, respectively. In some cases CS gave a significant P-value for a marker, while IA gave P ≥ 0.50 for the same marker. In all such instances, this was due to the fact that CS P-values for markers closely flanking the significant marker were high and not consistent with the low P-value of the significant marker. Thus, IA was more correctly reflecting the overall significance of the region. In these cases, the IA results were taken as definitive, and the single significant CS markers were attributed to technical error and therefore not taken as indicating a linked QTL. When both IA and CS indicated significance, the IA also provided a point estimate of QTL location according to the cM of highest significance, and a confidence interval of QTL location according to the region for which P < 0.05.

### Defining QTL containing regions (QTLR) and regions not containing QTL (non-Q regions)

Examination of the full set of marker test results showed that significant markers most often appeared in short stretches of two to five closely linked markers (in some cases incorporating one or more non-significant markers). These stretches of significant markers were flanked by stretches of non-significant markers. Each such stretch of one or more significant markers was taken to define a single QTL containing chromosomal region (QTLR). The flanking non-significant regions were taken to define regions that did not contain QTL (non-Q regions). The minimum extent of a QTLR is thus defined by the locations of the start and end markers of the QTLR; the maximum extent of a QTLR is defined as running from the midpoint between the distal marker of the QTLR and the proximal marker of the flanking upstream non-Q region to the midpoint between the proximal marker of the QTLR and the distal marker of the flanking downstream non-Q region. For example, QTLR 1–IV extended from 245 to 248 cM. It was flanked upstream by non-Q region 1–V having proximal marker at 259 cM, and flanked downstream by non-Q region 1–III having distal marker at 243 cM. Thus, the minimum extent of this QTLR was 3 cM, running from 245 to 248 cM; and the maximum extent of the QTLR was 9.5 cM, running from 244 cM to 253.5 cM.

### Estimating allele substitution effects

Allele substitution effects for a given QTLR were calculated as described in Appendix I, from the average differences, D, in allele frequency of the alleles derived from Line 2 in the resistant and susceptible pools. When a QTLR was represented by multiple markers in the same region, D was set equal to the weighted average D_ijk_-value across the resistant and susceptible pools of the 18 BF_jk _combinations within each of the M_i _markers defining the QTLR, weighted by the number of individuals in each pool.

### Searching for candidate genes in selected QTLR

The marker of interest for a specific QTL was identified in chicken genome assembly Build 2. Based on the cM per Kb from [45], the entire region was visualized on the USCS browser [46]. Using the USCS browser all putative genes in the region were identified using a variety of tools to visualize the features and annotations (e.g., chicken RefSeq, non-chicken RefSeq, chicken EST and mRNA). The identified genes were manually examined for a potential role in MD pathogenesis.

## Authors' contributions

EMH carried out all statistical analyses and participated actively in their interpretation and in writing all drafts of the manuscript. JEF participated in planning the study, carried out blood collections, DNA isolation, microsatellite genotyping and densitometric allele frequency analyses for selective DNA pooling and reviewed all drafts of the manuscript. NPO participated in planning the study and was responsible for implementing the crosses, directed the challenge tests, and provided overall coordination of the phenotyping aspects of the study. JAA was responsible for initiating and setting up this project, and providing initial overall direction. He participated actively in writing the final draft. HC added new SNP markers in selected regions of interest, carried out SNP genotyping, reviewed later drafts of the manuscript and carried out the candidate gene search. JW was involved in evaluating alternate mating designs for the later generations of the AIL, provided programs for the interval analysis and helped adjust them to the F6-AIL design. MS conceived and participated in planning the study and the later rounds of statistical analyses, and shared in their interpretation and in writing the advanced drafts of the manuscript. JCMD was involved in designing the later generations of the FSIL, secured funding for the statistical analysis of the data generated by the project, participated in planning and interpreting the statistical analysis and participated in writing the first draft and reviewing the later drafts of the manuscript.

All authors read and approved the final manuscript.

## Appendix 1

### Estimating the effects of individual markers on survival time

Let P_R _and P_S _be the proportion of total population selected to construct the resistant and susceptible pools; let α_P _be the observed allele substitution effect of the Line 2 marker allele relative to the Line 1 marker allele taken over both of the selected tails of the population; and let α_T _be the actual substitution effect in the population as a whole. Then, substituting in the Darvasi and Soller [31] expression, gives,

α_T _= α_P_/[(i_PR _+ i_PS_)/2]^2^,

where,

i_PX _= X_P_/P_X_, (P_X _= P_R _or P_S_) is the selection intensity of the pool,

X_P _is the ordinate of the standard normal distribution at the point Z_P _which cuts off proportion P of the distribution.

In the present study, P_S _= 0.22 was calculated from Table 1 as the total number of birds taken to the susceptible pools across all family and blood type combinations, including the Type II susceptible pools; P_R _= 0.44 was calculated from Table 1 as the weighted mean proportion selected across all family and blood type combinations with weighting according to the number of individuals in the pool.

With pool data, α_P _is calculated as

α_P _= G_2 _- G_1_

where, taking into account that an F6 population is equivalent to an F2 population with respect to expected genotype frequencies, and following Darvasi and Soller [31],

G_2 _is the mean of individuals homozygous for the Line 2 allele taken over all resistant and susceptible pools,

G_1 _is the mean of individuals homozygous for the Line 1 allele taken over all resistant and susceptible pools.

Letting, F_M2 _and F_M2S _be the frequency of the Line 2 allele (M2) in the resistant and susceptible pools, respectively, and F_M1R _and F_M1S _be the same for the Line 1 allele, then following Darvasi and Soller [31], relative frequency of G2 and G1 in the resistant pool (RF_G2R_, RF_G1R_) and in the susceptible pool (RF_G2S_, RF_G1S_) are estimated as

RF_G2R _= 2F_M2R_-0.5

RF_G2S _= 2F_M2S_-0.5

RF_G1R _= 2F_M1R_-0.5

RF_G1S _= 2F_M1S_-0.5

and,

G2 = RF_G2R _(T_R_) + RF_G2S_(T_S_)

G1 = RF_G1R _(T_R_) + RF_G1S_(T_S_)

Then, taking into account that

D = F_M2R _- F_M2S _= - (F_M1R _- F_M1S_),

we have with some algebra,

α_P _= G2 - G1 = 2D(T_R_-T_S_),

where

T_R _and T_S _are the mean survival time of the individuals in the R and S pools, respectively.

In the present study, since all individuals taken to the resistant pools survived until the end of the test, T_R _was taken as the mean test cut-off age across the two hatches, namely, 119.5 days; T_S _was taken as weighted mean survival time of the individuals in the susceptible pools, calculated from Table 1 across all family and blood type combinations = 63.02 days, with weighting according to the number of individuals in the pool. Thus, T_R _- T_S _= 56.5 days, and α_P _= 2D(56.5) days.

As noted in Heifetz et al. [18], when applied to survival data which have a right skewed distribution, the Darvasi and Soller [31] correction factor, which is based on the assumption of a normal distribution appears to provide estimates of QTL effect that are about 10% greater than the actual effects.
